# Ileosigmoid knotting: An update for Atamanalp classification

**DOI:** 10.12669/pjms.37.3.3179

**Published:** 2021

**Authors:** Sabri Selcuk Atamanalp

**Affiliations:** 1 Prof. Sabri Selcuk Atamanalp, MD, Department of General Surgery, Faculty of Medicine, Ataturk University, 25040, Erzurum, Turkey

**Keywords:** Ileum, Sigmoid Colon, Knotting, Classification, Treatment, Prognosis

## Abstract

Ileosigmoid knotting (ISK) is an extremely rare double-loop bowel obstruction. ISK is treated by emergency surgery with a relatively poor prognosis. Although some classification methods have been developed for ISK to date, the most comprehensive method was defined in 2018. Then, some subjects concerning this issue were evaluated in the literature. The aim of this paper is to update the last classification method in light of both the evaluations in the literature and our clinical experience with 80 ISK cases, which is one of the largest published single-centre ISK series.

## INTRODUCTION

Ileosigmoid knotting (ISK), the wrapping of the ileum or sigmoid colon around the base of the other structure causing a double-loop intestinal obstruction, was first described by Rokitansky in 1836.^1^ ISK is an extremely rare disease with a few hundreds cases reported to date.^2^ Many different classifications including prevalence (sporadic or endemic), active component (ileum or sigmoid colon), volvulus direction (counterclockwise or clockwise), and volvulus degree (<360, 360, or >360) have been published in literature.^3^ Nevertheless, none of the previous classification methods gives detailed information about the management or prognosis of ISK. In 2018, a new classification, treatment algorithm and prognosis-estimating system, in which age, American Society of Anesthesiologists (ASA) score, and the local bowel situation were used as therapeutic and prognostic data, was described for ISK.^3^ Following this description, some subjects concerning this issue were evaluated by some authors.^2^ In light of discussions mentioned above and our experience with 80 ISK cases treated over a 54-year period between June 1966 and July 2020, which is one of the largest published single-centre ISK series over the world,^2^ I thought about to update the present classification system.

## CLINICAL EXPERIENCE

I reviewed the records of 80 ISK cases retrospectively until June 1966 (56 patients) and prospectively thereafter (24 patients). An ethical approval was obtained via Institutional Ethics Committee (Ataturk University, Faculty of Medicine, 23.02.2017, B.30.2.ATA.0.01.00/17). In our series, 61 patients (76.3%) had gangrenous ileum and/or sigmoid colon. Of the patients, 16 (20.0%) were treated by surgical decompression and/or mesopexy, while ileum and/or sigmoid colon resection with primary anastomosis and/or stoma were performed in the remained 64 patients (80.0%). In this series, morbidity rate was 80.0% (16 patients), while mortality rate was 18.8% (15 patients).

## DISCUSSION

Despite modern anesthesia and surgery in addition to advanced perioperative care, the prognosis of ISK is still relatively poor.^4^,^5^ ISK requires emergency surgery following an early and effective resuscitation. In non-gangrenous patients, decompression alone is used or a volvulus preventing procedure such as colopexy, mesopexy or mesoplasty is added. In gangrenous cases, following the resection, primary anastomosis or stoma is preferred.^5^,^6^ Firstly, bowel viability and general condition of a patient are important factors in ISK.^7^,^8^ For this reason, I directly correlated the presence of bowel gangrene and poor ASA score with the management as well as the prognosis in my algorithm. Secondly, as it is well known, ISK is relatively common in undeveloped or developing countries.^1^,^4^ On the other hand, life expectancy, which is generally higher in developed countries, varies from a country to another one. Additionally, average lifetime decreases in time.^9^,^10^ For this reason, in my opinion, ‘life expectancy’ may be accepted as a checkpoint. As an example, I may accept the age limit as 75, which is mean life expectancy for men in Turkey, my practice area. Thirdly, adverse bowel conditions, including the presence of borderline ischemic, edematous or differantial scaled bowel ends, are important in decision-making process.^1^,^3^ Nevertheless, in my experience, viability, edema or diameter of the bowel ends must be evaluated following the resection, if needed. Finally, sigmoid volvulus, a component of ISK, may recur following a non-definitive surgery.^3^,^8^ For this reason, sigmoid resection may be suggested in some selected patients.

The results of our updated classification, which may have a role in a rapid decision for treatment and a correct estimation of prognosis are presented in Table-I

**Table I:** Atamanalp classification for ileosigmoid knotting.

Group	Definition	Surgical treatment	Mortality (%)	Morbidity (%)
I A	G 0, A 0, ASA I-III,	Decompression	1-5	5-15
		or plus sigmoid colopexy or mesopexy or mesoplasty	1-8	10-20
		or plus sigmoid resection and anastomosis	1-10	15-25
I B	G 0, A I or ASA IV-V	Decompression	10-30	20-40
II A	G I, A 0, ASA I-III, B 0	Ileum or sigmoid colon resection and anastomosis	5-20	10-30
II B	G I, A I or ASA IV-V or B I	Ileum or sigmoid colon resection and stoma	20-50	30-60
III A	G II, A 0, ASA I-III, B 0	Ileum and sigmoid colon resection and anastomosis	10-30	20-40
III B	G II, A I or ASA IV-V or B I	Ileum and sigmoid colon resection, one anastomosis and one stoma	30-60	40-80

A 0, age < life expectancy; A I, age ≥ life expectancy; ASA I, patient with no other disease; ASA II, patient with mild systemic disease; ASA III, patient with severe systemic disease; ASA IV, patient with life-threating systemic disease; ASA V, moribund patient; B 0, normal bowel; B I perforated bowel or borderline ischemic, edematous or differential scaled bowel ends following resection; G 0, viable bowel, G I, gangrenous sigmoid colon or ileum; G II, gangrenous double segment.

## CONCLUSION

In ISK, when the diagnosis and resuscitation were made, the usage of the present upgraded classification method may help the distribution of an ISK patient into groups in 30 seconds, the decision for the management option in 15 seconds, and a comment about the prognosis in the subsequent 15 seconds. Thus wise, the theoretical evaluation may be completed in approximately a minute and the surgical treatment may be applied in a short time.
